# Exploring the Multifaceted Influences on Childhood Nutritional Status: A Study Conducted in South Punjab, Pakistan

**DOI:** 10.7759/cureus.64329

**Published:** 2024-07-11

**Authors:** Aiman Akhtar, Rabiya Masood, Muhammad Ibrahim, Neelab Gurmani, Muhammad Abdullah, Abdullah Ali, Talha Kareem

**Affiliations:** 1 National Institute of Health, Health Services Academy, Islamabad, PAK; 2 Community Medicine, Nishtar Medical University, Multan, PAK; 3 Medicine, Al Abid Clinic, Rawalpindi, PAK; 4 Preventive Medicine, Integrated Reproductive, Maternal, Newborn and Child Health (IRMNCH) Program, Multan, PAK; 5 Surgery, Shifa College of Medicine, Shifa Tameer-E-Millat University, Islamabad, PAK; 6 General Surgery and Vascular Surgery, Indus Hospital, Indus Hospital and Health Network, Muzaffargarh, PAK; 7 General Surgery, Nishtar Medical University, Multan, PAK

**Keywords:** punjab (pakistan), multan, bmi, dietary habits, socioeconomic factors, children, underweight, overweight

## Abstract

Introduction

The double burden of malnutrition (DBM) in Pakistan is a rising concern affecting school-going children, marked by coexisting under- and over-nutrition within the same population. Key influences include shifts in dietary habits, socioeconomic status, and lifestyle changes due to rapid urbanization. With a focus on Multan, Pakistan, the study seeks to assess the proportion of underweight and overweight students while identifying the risk factors and sociodemographic characteristics associated with this incidence. The aim is to guide future health interventions addressing this multidimensional health challenge.

Materials and methods

This study, adopting a descriptive cross-sectional research design, collected data from female teenage students through interviews and anthropometric measurements. A total of 300 participants were randomly selected from a comprehensive school list representing diverse urban and rural settings. Participants' weight and height were measured to calculate their body mass index (BMI), categorizing them into underweight, normal weight, and overweight groups. The relevant risk factors were collected through an interview questionnaire. Collected data were analyzed using IBM SPSS Statistics for Windows, Version 26.0 (Released 2019; IBM Corp., Armonk, New York, United States), with the results stratified according to socioeconomic, dietary, and psychosocial factors and compared across different weight categories.

Results

The study collected data from 300 students, revealing a correlation between socioeconomic status, dietary habits, and BMI. Parental occupation significantly affected nutritional status, with children of laborers primarily falling within normal and underweight categories. Dietary habits like frequency of fast food and milk or dairy consumption showed notable associations with nutritional status. Psychosocial factors such as peer or teacher comments about weight and outdoor sports participation also influenced the students' nutritional status. However, factors like family income, video game hours, and the presence of pets at home did not show significant associations with nutritional status.

Conclusions

The study illustrates a multi-faceted association between socioeconomic status, dietary habits, and BMI among schoolchildren in Multan, Pakistan, emphasizing the need for comprehensive interventions.

## Introduction

The double burden of malnutrition, encompassing both undernutrition and overnutrition, is prevalent in low- and middle-income countries, notably in regions such as sub-Saharan Africa, South-East Asia, and the Pacific [1]. Despite global efforts to combat malnutrition, progress remains slow. In developing countries, particularly, the issue is intricately linked with poverty, affecting children from low-income families disproportionately. Factors contributing to this complex problem include insufficient food intake, poor health conditions, and limited access to education and food resources [2].

Transitioning economies, especially in developing nations, witness a concurrent rise in chronic undernutrition and obesity. This phenomenon, known as the double burden of malnutrition (DBM), has shifted its prevalence towards low-income quartile nations, highlighting the evolving nature of malnutrition challenges [3]. Factors such as changes in dietary patterns, increased availability of unhealthy food, and reduced physical activity due to technological advancements contribute to the rise in overweight individuals, particularly in urban areas [4].

In countries like Pakistan, the dual burden of malnutrition poses significant health risks, particularly for children. Undernutrition, often exacerbated by poverty and inadequate healthcare access, stunts growth, weakens immune systems, and hinders cognitive development. Conversely, overnutrition, fueled by urbanization and dietary shifts towards processed foods, escalates the risk of non-communicable diseases, further straining healthcare systems and exacerbating socioeconomic disparities [5].

In the context of Pakistan, understanding the prevalence of underweight and overweight students, along with sociodemographic determinants, is crucial for targeted interventions. By identifying high-risk groups and addressing the root causes of malnutrition, effective strategies can be developed to mitigate the double burden of malnutrition and promote healthier outcomes for children.

Globally, childhood obesity rates have reached alarming levels, fueled by shifts in dietary patterns and sedentary lifestyles. In Pakistan, despite historical undernutrition challenges, there's a significant rise in childhood obesity, especially in urban areas. Studies indicate a concerning increase in overweight and obesity rates among school-aged children, emphasizing the need for multifaceted interventions addressing dietary habits, physical activity levels, and socioeconomic factors [6-9].

Undernutrition remains a pervasive issue in Pakistan, with millions of children suffering from stunting and deficiencies in essential nutrients. Limited access to nutritious food, inadequate maternal and child healthcare, and poor dietary practices contribute to the prevalence of undernutrition. While efforts have been made to address undernutrition, the emergence of the double burden of malnutrition underscores the need for holistic approaches that tackle both undernutrition and overnutrition effectively [10-12]

Several factors contribute to the rising rates of overweight and undernutrition among Pakistani children. Sedentary behaviors, poor dietary choices, and socioeconomic disparities play significant roles in shaping children's nutritional status. Studies highlight the importance of addressing these factors through targeted interventions, including promoting healthy lifestyles, improving access to nutritious food, and raising awareness about the risks of unhealthy dietary habits [13-16].

Addressing the double burden of malnutrition requires a multifaceted approach involving various sectors. Strategies such as improving access to nutritious food, promoting healthy dietary practices, and strengthening healthcare systems are essential for mitigating malnutrition challenges. By prioritizing the well-being of children and implementing evidence-based interventions, Pakistan can work towards achieving better health outcomes and reducing the burden of malnutrition in the country [17,18].

## Materials and methods

This was a descriptive cross-sectional study conducted in Multan, Pakistan, from May 2023 to July 2023. The study was approved by the Indus Health Network Institutional Review Board (approval number: IHHN_IRB_2022_12_024). Permission was secured from school authorities, and verbal consent was obtained from participants. Confidentiality was ensured, and interviews were conducted during recess to minimize disruption.

The participants included teenagers aged 12-15 years from elementary (grades 4-5) and middle (6-8) schools in Multan, Punjab region, Pakistan. Children with mental illness or insufficient cognitive ability, severe chronic illness, or those unwilling to participate were excluded from the study.

Data collection

Eight schools from the Multan district (enlisted by the Punjab School Education Department) were randomly selected and included in this study. The height and weight of students were measured, and BMI was calculated for each student. Interviews were conducted by medical students during recess, with the option for students to choose whether their names were included on the form. The interviews were conducted using a questionnaire detailed in the Appendices.

Sampling size

The minimum sample size was estimated to be 196, based on a confidence level of 95%, an anticipated population proportion of overweight at 15% [19], and an absolute precision error of ±5%. Gender differences were not considered in this calculation. All consenting students within the selected grades and schools were included to ensure comprehensive representation. Simple random sampling was used. Students between the ages of 12 and 15 years in the seven selected schools were randomly selected. 

Variables

Data were collected on various domains including socioeconomic factors, family health and history, dietary habits, and social and psychological factors, categorized into numerical and categorical variables.

Data analysis

Data were analyzed using IBM SPSS Statistics for Windows, Version 26.0 (Released 2019; IBM Corp., Armonk, New York, United States). Descriptive statistics were used for numerical variables, while ANOVA and Chi-Square test of Independence were employed for comparisons between BMI-based weight categories. p-value < 0.05 was considered significant.

## Results

Data from 300 students were gathered through interviews conducted across multiple schools. Table 1 presents their demographic details. The participants had an average age of 13.74 ± 1.13 years, and their BMI averaged 20.66 (SD = 7.26). Labor-intensive occupations, notably laborers, accounted for 147 (49.15%) of parental occupations, with 195 (65%) of children belonging to the lower socioeconomic class. About 12 (4%) had chronic illnesses and 92 (32%) had personal histories of chronic illnesses. In terms of dietary habits, children consumed an average of 2.75 ± 0.82 meals per day, with 299 (99.7%) reporting frequent meal skipping (Table 2). Vegetable consumption was prevalent (99.7%), while fast food consumption was common (168 (56%) consuming less than twice a week and 132 (44%) consuming more). Approximately 168 (56% ) reported regular consumption of cookies and/or ice cream, and 111 (37%) regularly consumed milk or dairy products. Socially, children engaged in limited outdoor sports (0.98 hours per week) and spent more time playing video games (1.89 hours per week). Additionally, about 74 (24.7%) of children faced weight-related comments from peers or teachers.

**Table 1:** Demographic characteristics of the respondents (N=300) The data has been represented as n (%) and mean±SD

Variable	Values
Age (years), mean±SD	13.74 ± 1.13
BMI (kg/m^2^), mean±SD	20.66 ± 7.26
Occupation of Parent, n (%)	
Unemployed	9 (3.05%)
Farmer	45 (14.92%)
Government/private job	46 (15.25 %)
Labourer	147 (49.15%)
Shopkeeper/business	30 (9.83%)
Trade jobs	23 (7.8%)
Family Income, mean±SD	33796.67 ± 30703.06
Socioeconomic Status, n (%)	
Upper class	1 (0.33%)
Middle class	104 (34.67%)
Lower class	195 (65%)

**Table 2:** Health and family history, dietary habits, and social and psychological characteristics of the respondents (N=300) The data has been represented as n (%) and mean±SD

Characteristics	Values
Health and Family History	
Presence of any chronic illness, n (%)	12 (4.0%)
Dietary Habits	
Number of meals per day, mean±SD	2.75 ± 0.82
Occasional skipping of meals, n (%)	299 (99.7%)
Frequency of meat consumption per week, mean±SD	2.12 ± 1.05
Regular consumption of vegetables	299 (99.7%)
Fast food consumption, n (%)	
Less than twice a week	168 (56%)
More than twice a week	132 (44%)
Regular consumption of cookies and/or ice cream, n (%)	168 (56%)
Regular consumption of coffee or tea, n (%)	68 (22.7%)
Frequency of milk or dairy product consumption, n (%)	111 (37%)
Social and Psychological	
Weekly hours of playing outdoor sports, mean±SD	0.98 ± 0.99
Weekly hours of playing video games, mean±SD	1.89 ± 0.91
Presence of pets at home, n (%)	26 (8.7%)
Peers/teachers' comments on weight, n (%)	74 (24.7%)
The time gap between the last meal and bedtime, mean±SD	8.93 ± 0.70

Among the 300 participants, 158 (52.7%) were underweight, 80 (26.7%) had a normal BMI, and 62 (20.7%) were overweight. They were categorized based on weight, and comparisons were drawn to analyze the impact of socioeconomic, family health, dietary habits, and socio-psychological factors. Socioeconomic factors are seen to significantly affect nutritional status (Table 3). Age (p = 0.022) and BMI (p < 0.001) were significantly correlated with nutritional status. Parental occupation showed a significant impact (p < 0.001), with laborers' children mostly underweight or normal, while farmers' children were often overweight. Family income didn't correlate significantly (p = 0.778), but socioeconomic status did (p < 0.001), with lower classes having more underweight individuals and upper classes fewer, emphasizing socioeconomic influence on nutrition.

**Table 3:** Effect of socioeconomic factors on nutritional status The data has been represented as n (%), mean±SD; Chi Square test was used in case of categorical variables; ANOVA was used in case of continuous variables * p-value < 0.05 is considered significant

Variable	Under-weight (n=158)	Normal (n=80)	Over-weight (n=62)	p-value
Age (years)	13.77±1.2	13.94±1.1	13.42±1	0.022*
BMI (kg/m^2^)	16.53±2.4	19.74±2.6	32.36±7.1	<0.001*
Occupation of Parent				
Unemployed				<0.001*
Yes	1 (0.6%)	1 (1.3%)	7 (11.3%)	
No	157 (99.4%)	79 (98.7%)	55 (88.7%)	
Farmer				
Yes	19 (12.1%)	2 (2.6%)	23 (37.1%)	
No	139 (87.9%)	78 (97.4%)	39 (62.9%)	
Government/private job				
Yes	34 (21.7%)	7 (9.2%)	4 (6.5%)	
No	124 (78.3%)	73 (90.8%)	58 (93.5%)	
Labourer				
Yes	73 (46.5%)	59 (77.6%)	13 (21%)	
No	85 (53.5%)	21 (22.4%)	49 (79%)	
Shopkeeper/business				
Yes	15 (9.6%)	6 (7.9%)	8 (12.9%)	
No	143 (90.4%)	74 (92.1%)	54 (87.1%)	
Trade jobs				
Yes	15 (9.6%)	1 (1.3%)	7 (11.3%)	
No	143 (90.4%)	79 (98.3%)	55 (88.7%)	
Family Income	34930.38±38903.3	33037.5±21130.36	31887.1±11803.17	0.778
Socioeconomic Status				
Upper class				<0.001*
Yes	1 (0.6%)	0 (0%)	0 (0%)	
No	157 (99.4%)	80 (100%)	62 (100%)	
Middle class				
Yes	55 (34.8%)	13 (16.3%)	36 (58.1%)	
No	103 (65.2%)	67 (83.7%)	26 (41.9%)	
Lower class				
Yes	102 (65%)	67 (83.8%)	26 (41.9%)	
No	56 (35%)	13 (16.2%)	36 (58.1%)	

Table 4 highlights factors related to participants' nutritional status. The number of siblings was significantly associated (p < 0.001) with nutritional status, with underweight individuals having more siblings. Chronic illness presence didn't significantly correlate (p = 0.156), but personal history did (p < 0.001), especially in overweight individuals. Siblings' chronic illness presence (p = 0.637) and their weight status (p = 0.561) weren't significantly associated with nutritional status.

**Table 4:** Effect of health and family history on nutritional status The data has been represented as n (%) and mean±SD. *p-value < 0.05 is considered significant.

Variable	Under-weight (n=158)	Normal (n=80)	Over-weight (n=62)	p-value
Number of siblings	4.89±1.93	4.13±1.50	3.42±1.87	<0.001*
Presence of any chronic illness				
Yes	7 (4.4%)	5 (6.3%)	0 (0%)	0.156
No	151 (95.6%)	75 (93.7%)	62 (100%)	
Siblings with chronic illness				
Yes	1 (0.6%)	0 (0%)	0 (0%)	0.637
No	157 (99.4%)	80 (100%)	62 (100%)	
Siblings overweight or underweight				
Yes	156 (98.7%)	80 (100%)	61 (98.4%)	0.561
No	2 (1.3%)	0 (0%)	1 (1.6%)	

Table 5 demonstrates the impact of dietary habits on participants' nutritional status. The number of meals per day (p < 0.001) and meat consumption (p < 0.001) correlated significantly with nutritional status. While skipping meals was common, it didn't significantly relate to nutritional status (p = 0.146). Vegetable consumption showed no significant association (p = 0.146). Notably, fast food consumption (p < 0.001) was significantly linked to overweight status and cookies/ice cream consumption (p < 0.001) correlated significantly with underweight status. Coffee/tea consumption had a significant correlation (p = 0.005) with normal weight, and milk/dairy consumption (p < 0.001) was associated with overweight status.

**Table 5:** Effect of dietary habits on nutritional status The data has been represented as n (%) and mean±SD. *p-value < 0.05 is considered significant

Variable	Under-weight (n=158)	Normal (n=80)	Over-weight (n=62)	p-values
Meals per day	2.63±0.72	3.2±0.72	2.47±0.95	<0.001*
Skipping of meals				
Yes	158 (100%)	80 (100%)	61 (98.4%)	0.146
No	0 (0%)	0 (0%)	1 (1.6%)	
Meat consumption per week	2.22±1.08	2.45±0.9	1.45±0.84	<0.001*
Consumption of vegetables				
Yes	158 (100%)	80 (100%)	61 (98.4%)	0.146
No	0 (0%)	0 (0%)	1 (1.6%)	
Fast food				
< twice a week	118 (74.7%)	39 (48.8%)	11 (17.7%)	<0.001*
> Twice a week	40 (25.3%)	41 (51.3%)	51 (82.3%)	
Cookies and/or ice cream				
Yes	95 (60.1%)	59 (73.8%)	14 (22.6%)	<0.001*
No	63 (39.9%)	21 (26.2%)	48 (77.4%)	
Coffee or tea				
Yes	24 (15.2%)	25 (31.3%)	19 (30.6%)	0.005*
No	42 (84.8%)	55 (68.7%)	43 (69.4%)	
Milk or dairy				
Yes	70 (44.3%)	12 (15%)	29 (46.8%)	<0.001*
No	88 (55.7%)	68 (85%)	33 (53.2%)	

**Figure 1:**
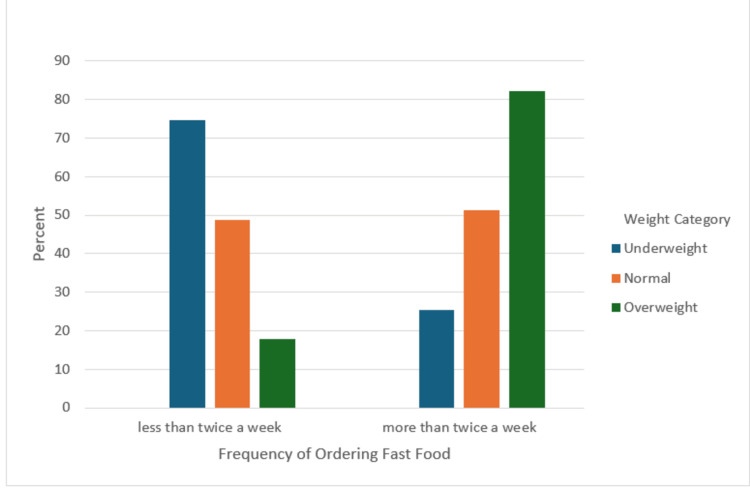
Association of fast-food eating frequency with the different weight categories

Figure 1 shows the relation between fast food eating frequency and weight categories. 

Table 6 examines social and psychological factors related to participants' nutritional status. Weekly hours of outdoor sports correlated significantly (p = 0.001) with nutritional status, with underweight individuals spending fewer hours outdoors. However, weekly hours of video game play showed no significant association (p = 0.483). The presence of pets at home (p = 0.22) and the time gap between the last meal and bedtime (p = 0.771) did not show significant associations. Notably, peers'/teachers' weight-related comments significantly correlated (p = 0.008) with underweight status.

**Table 6:** Effect of social and psychological factors on nutritional status The data has been represented as mean ±SD. *p-value < 0.05 is considered significant

Variable	Under-weight (n=158)	Normal (n=80)	Over-weight (n=62)	p-value
Weekly hours of playing outdoor sports	1.08±1.14	1.11±0.73	0.55±0.69	0.001*
Weekly hours of playing video games	1.88±0.92	1.99±0.99	1.81±0.77	0.483
Pets at home				
Yes	20 (12.7%)	5 (6.3%)	1 (1.6%)	0.22
No	130 (87.3%)	75 (93.7%)	61 (98.4%)	
Peers/teachers' comments on weight				
Yes	40 (25.3%)	27 (33.8%)	7 (11.3%)	0.008*
No	118 (74.7%)	53 (66.2%)	55 (88.7%)	
The time gap between the last meal and bedtime	8.91±0.79	8.91±0.78	8.98±0.13	0.771

## Discussion

In our study, the majority of participants (52.7%) were underweight. Numerous studies evaluating the nutritional condition of children from various population segments have been carried out [11,15]. The majority of the 634 child laborers in a study by Meesha et al. were underweight (stunted or wasted) [20]. However, according to other studies, the majority of students attending wealthy schools in Dhaka were overweight or obese [7]. Our research revealed a strong relationship between parental occupation and socioeconomic class and the nutritional status of children. In our study, the majority of overweight participants were from middle-class backgrounds, whereas the majority of underweight participants came from lower-class backgrounds. An additional study, a review paper by Christian et al. also found that there is a connection between childhood obesity and socioeconomic class [21]. Children who had inadequate nutrition at a young age are more likely to be obese as children and to have other nutritional deficits; these children are also more likely to come from low-income homes [22,23]. In a similar vein, Nuwabese et al.'s study discovered a statistically significant relationship between parental education and the incidence of underweight children [24]. Research has also indicated that maternal education helps children's nutritional health [25].

Our study showed that unhealthy dietary practices like eating fast food, energy-dense foods, and an increased number of meals were associated with conditions like obesity. Literature also shows that dietary behaviors such as frequent consumption of fast food and sweetened beverages were associated with overweight or obesity, reflecting global trends in childhood nutrition [26]. While dairy consumption is often perceived as beneficial, our findings suggest that excessive intake may contribute to overweight status, emphasizing the importance of moderation.

Results of our study show that social and psychological factors play significant roles in childhood nutritional status. A possible explanation of the association between the number of siblings and nutritional status are challenges faced by children from larger families in accessing adequate nutrition and care [27]. Our study also shows that limited outdoor physical activity further exacerbates the risk of overweight and obesity. Many studies have been conducted that have identified low levels of physical activity as a risk factor for childhood obesity [28].

The results showed that weight-related comments from peers or teachers significantly correlated with underweight status. Rebecca et al. conducted a 15-year longitudinal study that showed weight-based teasing resulted in unhealthy dietary habits and emotional ill-being resulted in weight disorders [29]. These findings underscore the importance of addressing social and psychological factors in interventions aimed at improving childhood nutritional health.

Limitations and strengths of the study

Female teenage girls were only included in the study which decreased the generalizability of the findings to the entire population of school-going children in Multan. The study was conducted in Multan so the results might not be the same for other rural or urban areas of Pakistan. Further studies are needed in those areas to evaluate the knowledge, attitudes, and practices of students.

The study's strength lies in its focus on the double burden of malnutrition, a critical and escalating health issue in Pakistan, particularly among school-going children. By examining a wide range of socio-demographic, dietary, and psychosocial factors, the study provides a thorough understanding of the influences on children's nutritional status. This comprehensive approach has direct implications for designing targeted health interventions, as it identifies key areas for effective intervention and policy-making.

## Conclusions

The findings of this study highlight the multifaceted nature of childhood nutritional status in Multan, Pakistan. Socioeconomic conditions, parental occupation, and family status were identified as significant contributors to being underweight and overweight. Dietary habits, including fast food and dairy product consumption, along with meal frequency, were also linked to variations in nutritional status. Sociopsychological factors, such as the number of siblings and levels of outdoor physical activity, emerged as crucial determinants.

This study underscores the intricate interplay between various factors influencing childhood nutritional health, emphasizing the need for comprehensive, multidimensional strategies to address nutritional disorders in the region. Future research should focus on identifying precise causal mechanisms and monitoring the long-term effects of these factors on childhood nutritional health to inform targeted interventions effectively.

## Appendices

Data collection form

Dietary, Social, and Psychological Section

**Table 7:** Questionnaire: dietary, social, and psychological section

Dietary, social, and psychological factors	
Number of meals per day	
Do you occasionally skip meals	Yes No
How sure are you that you will get your next	Very sure Not sure
Do you regularly eat vegetables in your diet	Yes No
How many times a week do you eat meals	
How many hours a week do you play video games	
How many hours a week do you play outside	
Do any of your siblings suffer from any chronic illness	Yes No
Were any of your siblings overweight or underweight	Yes No
Are both of your parents working	Yes No
How often do you eat out: Less than twice a week; More than twice a week	Less than two times a week; More than two times a week No
Did you have any chronic illness as a child	Yes No
Do you eat ice cream and cookies regularly	Yes No
Do your peers and teachers call you overweight or underweight	Yes No
At what time do you wake up and at what time do you sleep	
How many hours do you generally sleep	
Do you take coffee or tea regularly	Yes No
Do you regularly take milk and dairy products	Yes No

Demographics Section

**Table 8:** Questionnaire demographics section

Demographics	
Registration number	
Age	
BMI	
Occupation of Parent	
	Unemployed
	Farmer
	Gov/private job
	Labourer
	Shopkeeper/business
	Trade jobs
Number of siblings	
History of chronic illness	
Family Income	
Residential area	
Socioeconomic Status	
	Upper class
	Middle class
	Lower class
